# Circulating adiposity‐related microRNAs as predictors of the response to a low‐fat diet in subjects with obesity

**DOI:** 10.1111/jcmm.14920

**Published:** 2020-01-22

**Authors:** Taís Silveira Assmann, José I. Riezu‐Boj, Fermín I. Milagro, J. Alfredo Martínez

**Affiliations:** ^1^ Department of Nutrition, Food Science and Physiology Center for Nutrition Research University of Navarra Pamplona Spain; ^2^ Centro de Investigación Biomédica en Red Fisiopatología de la Obesidad y Nutrición (CIBERobn) Instituto de Salud Carlos III Madrid Spain; ^3^ IdiSNA Navarra Institute for Health Research Pamplona Spain; ^4^ Madrid Institute of Advanced Studies (IMDEA Food) Food Institute Madrid Spain

**Keywords:** biomarkers, dietary intervention, microRNAs, obesity, weight loss

## Abstract

Recent studies have revealed the critical role of several microRNAs (miRNAs) in energy homeostasis and metabolic processes and suggest that circulating miRNAs can be used as early predictors of weight loss in the design of precision nutrition. Thus, the aim of this study was to investigate circulating adiposity‐related miRNAs as biomarkers of the response to two specific weight loss dietary treatments. The expression of 86 miRNAs was investigated in plasma of 78 subjects with obesity randomized to two different diets [moderately high‐protein diet (n = 38) and low‐fat diet (n = 40)] and in 25 eutrophic controls (BMI ≤ 25 kg/m^2^). Bioinformatic analyses were performed to explore the target genes and biological pathways regulated by the dysregulated miRNAs. As results, 26 miRNAs were found differently expressed in eutrophic and volunteers with obesity. Moreover, 7 miRNAs (miR‐130a‐3p, miR‐142‐5p, miR‐144‐5p, miR‐15a‐5p, miR‐22‐3p, miR‐221‐3p and miR‐29c‐3p) were differentially expressed between responders and non‐responders to a low‐fat diet. Furthermore, after adjustment for basal glucose levels, 1‐SD increase in miR‐22‐3p expression was associated with reduction in the risk of non‐response to low‐fat diet [OR = 0.181, 95% CI (0.084‐0.947), *P* = .043]. Bioinformatic analyses evidenced that these 7 miRNAs regulate the expression of genes participating in important metabolic pathways. Conclusively, 7 circulating miRNAs related to adiposity could be used for predicting the response to a low‐fat diet intervention prescribed to lose weight.

## 1 INTRODUCTION

Obesity is a worldwide epidemic that arises as a chronic long‐term imbalance between calorie intake and energy expenditure.^1^ Despite nutritional interventions and physical education programmes, the obesity prevalence is still increasing and ∼600 million people worldwide are expected to have obesity by 2025.^1^ In this context, common behavioural strategies for weight management and reducing fat mass comprise dietary energy restriction and increased energy expenditure through physical activity,^2^ although there is a great variability in the inter‐individual response to weight loss, according to the strategy used.^3^, ^4^, ^5^ While the adherence to the prescribed diet and physical activity questionless contributes to variation in fat loss among individuals, differences in genotype may also be a determinant of fat loss during any weight loss intervention.^3^, ^4^ Additionally, this variability in the weight loss response may also be attributed to epigenetic factors modulated by behavioural/environmental aspects.^3^, ^6^, ^7^

Among the epigenetic mechanisms, microRNAs (miRNAs) are a class of small non‐coding RNAs that regulate gene expression.^8^, ^9^ These molecules have well‐known roles in the regulation of several biological processes, including cellular differentiation, proliferation, metabolism, ageing and apoptosis.^9^ Additionally, it is estimated that the miRNAs regulate the expression of more than 60% of protein‐coding genes 8; thus, changes in miRNAs expression have been associated with several diseases, such as metabolic disorders and obesity.^10^, ^11^

While some miRNAs are highly expressed in various tissues throughout the body, a subgroup of miRNAs are found circulating in biological fluids.^12^ Some of them may be transported in an active form to other tissues, increasing the possibility of miRNA involvement in a cell‐to‐cell communication mode.^3^, ^4^, ^12^ Furthermore, the circulating miRNAs profile is different between subjects with obesity and eutrophic individuals.^13^ More interestingly, in the pursuit of the identification of potential miRNAs as weight loss biomarkers, a differential abundance of specific miRNAs has been reported in peripheral blood mononuclear cells when comparing high and low responders following an 8‐week energy‐restricted diet.^5^ Moreover, only one study has previously analysed this type of molecules in plasma samples, describing that the expression of miR‐935 was higher in low responders compared to high responders to a 16‐week intervention during which an energy deficit of 500 kcal was induced through an energy‐restricted diet (~250 kcal) and daily exercise (~250 kcal).^14^

With this background, the objective of the present research was to investigate whether the expression of circulating adiposity‐related miRNAs can be useful in the prediction of individual response to two specific 16‐week weight loss interventions, in order to implement this type of biomarkers in the personalization of obesity treatment. A second goal was to determine whether the circulating miRNA signature before the weight loss intervention is associated with the intervention‐induced changes in anthropometric, lipid or glucose parameters. Additionally, bioinformatics analyses were performed to investigate the target genes and biological pathways regulated by the miRNAs potentially associated with weight loss.

## 2 METHODS

### 2.1 Study population

This study was developed according to STROBE guidelines to report association studies.^15^ The sample included 78 subjects with obesity (BMI: 30‐40 kg/m^2^) who were randomized to two different diets: diet 1 (moderately high‐protein diet, n = 38) and diet 2 (low‐fat diet, n = 40). Moreover, a group of 25 healthy individuals was also included (BMI ≤ 25 kg/m^2^).

Obesity was classified following the World Health Organization guidelines.^1^ All volunteers were enrolled from October 2015 to February 2016 in the Metabolic Unit of the Centre for Nutrition Research of the University of Navarra, Spain. Major exclusion criteria were as follows: history of diabetes mellitus, cardiovascular disease or hypertension, pregnant or lactating women, current use of lipid‐lowering pills or medications that affect body weight, and weight change ≥ 3 kg within 3 months before the recruitment.

This study followed the ethical principles for medical research in humans from the Declaration of Helsinki, 2013.^16^ Furthermore, the research protocol was approved by the Research Ethics Committee of the University of Navarra (ref. 132/2015) and registered at clinicaltrials.gov (reg. no. NCT02737267). A written informed consent of each participant was obtained prior to enrolment in the study.

### 2.2 Dietary intervention

Energy requirements were individually assessed from resting energy expenditure according to the Mifflin formula, multiplied for physical activity level calculated by a short 24‐hour physical activity questionnaire.^17^ Diets had the following macronutrient composition: (a) *moderately high‐protein diet*: 40% of total energy from carbohydrates, 30% from protein and 30% from fat; and (b) *low‐fat diet*: 60% of total energy from carbohydrates, 18% from protein and 22% from fat. Prescribed diets led to a restriction of 30% of the total energy expenditure estimated for each individual. No initial prescribed diets had less than 1200 kcal/d. The intervention period lasted a total of 16 weeks. The adherence to the diets was graded by trained dietitians during each intervention visit according to the following scale: 0: failure to follow the diet at any time (poor adherence); 1: follow‐up across weekdays but not during weekends (regular adherence); 2: occasionally exceeded from recommendations (good adherence); and 3: continuous follow‐up (very good adherence), as described elsewhere.^18^ Only subjects with regular to very good adherence were included. This test was applied twice, in the middle (8th week) and at the end (16th week) of the intervention. In addition, the real macronutrient distribution of both diets was monitored by means of food records of 3 days (including 2 weekdays and 1 day of the weekend), which were applied at two times, in the middle and at the end of the intervention.^18^

Individuals were randomly assigned to one of the two diets by a logarithm design for this study by MATLAB using stratified block randomization. Compliance analysis to the recommended diet of the participants was performed considering the three‐day‐weighed food record at two times: in the middle and at the end of the intervention period. Total energy consumption and nutrient content were determined using validated Spanish food composition tables and appropriate software.^19^

### 2.3 General measurements

All volunteers underwent conventional anthropometric and laboratory measurements, as described elsewhere.^20^ The measurements of height, body weight and waist circumference were collected at the fasting state by trained nutritionists following validated procedures.^20^ BMI was calculated as the ratio between weight and squared height (kg/m^2^). Body composition was quantified using dual‐energy X‐ray absorptiometry according to the instructions provided by the manufacturer (Lunar Prodigy, software v6.0,). Biochemical measurements including glycemic and lipid profiles were performed using an automatic analyser (Pentra C200, HORIBA Medical). Insulin, adiponectin and leptin were quantified with commercial ELISA kits (Mercodia Insulin ELISA, Biovendor Human adiponectin ELISA and Mercodia Leptin).

Insulin resistance was investigated by the homeostatic model assessment‐insulin resistance (HOMA‐IR) index according to the following formula: (fasting insulin (mU/L) × plasma glucose (mmol/L)/22.5), while the triglyceride‐glucose (TyG) index was calculated as: ln [fasting triglycerides (mg/dL) × fasting plasma glucose (mg/dL)/2], as previous described.^21^

The habitual consumption (daily, weekly, monthly or never) of 137 foods during the previous year was assessed using a validated semiquantitative food frequency questionnaire.^22^ Energy and nutrient intakes were calculated using an ad hoc computer program based on the standard Spanish food composition tables.^19^ The physical activity level was estimated using a validated questionnaire,^23^ and the volume of activity was expressed in metabolic equivalents (METs).^24^

### 2.4 MicroRNA expression analysis

#### 2.4.1 Search for miRNAs related to adiposity and obesity

The selection of the 86 target miRNAs related to adiposity and weight loss was based on available literature 11, 25 and also on the search for miRNAs potentially associated with obesity in humans using the miRWalk 2.0 database.^26^

#### 2.4.2 miRNA isolation and reverse transcription‐quantitative PCR

Total RNA was isolated from 200 μL EDTA‐plasma using the miRNeasy Serum/Plasma Advanced kit (Qiagen) in accordance with the manufacturer's recommendations. RNA spike‐in was added to each sample. The purity and concentration of RNA samples were measured using the NanoDrop ND‐1000 Spectrophotometer (Thermo Fisher Scientific). Only RNA samples with satisfactory purity ratios (A260/A280 = 1.9‐2.1) were included in the subsequent analyses.^27^

**Figure 1:**
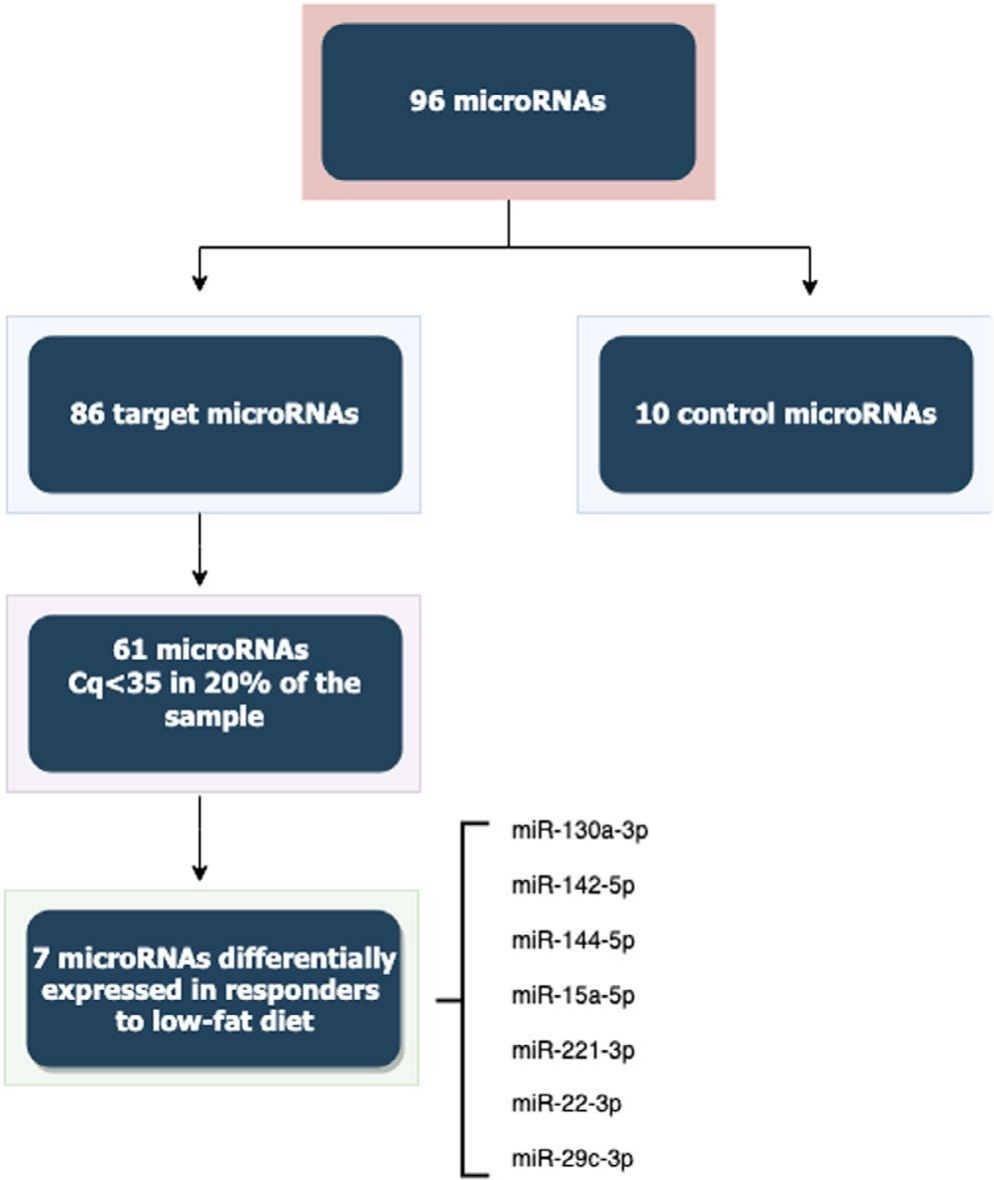
Flow chart showing the selection of miRNA for the present study. From 86 target miRNA, 25 were eliminated due to low expression in more than 80% of samples. Cq, quantification cycle, miRNA, microRNA

The relative expression of the 86 target miRNAs was analysed in plasma from all individuals using Custom Pick‐&‐Mix microRNA PCR Panel v5 (Qiagen). Moreover, 9 controls (reference genes + Spike‐in controls) and a blank were also included in each plate, as shown in Figure 1 and Table S1. Total RNA (4 μL) was reverse‐transcribed in 10 μL reaction using the miRCURY LNA Universal RT microRNA PCR, Polyadenylation and cDNA synthesis kit II. RT‐qPCR experiments were done using a CFX384 Real‐time system (Bio‐Rad). The following cycling conditions were used: 95°C for 2 minutes, followed by 40 cycles at 95°C for 10 seconds and 56°C for 1 minute. Relative expressions were calculated using the 2^−ΔΔCt^ method.^28^

#### 2.4.3 Quality control and normalization

Quality control was carried out using synthetic spike‐in RNAs to analyse the robustness of the RNA isolation process and quality of extracted miRNA. The RNA isolation controls (UniSp2, UniSp4 and UniSp5; Exiqon, Denmark) were added to the thawed plasma before the isolation process, aiming to detect differences in the extraction efficiency. The cDNA synthesis control (UniSp6, Exiqon) and cel‐miR‐39‐3p were added to the reverse transcription reaction to determine the effectiveness of this process. Furthermore, the UniSp3 was used as inter‐plate calibrator and PCR amplification control, included in all plates.

Haemolysis was measured by the ratio between hsa‐miR‐451a and hsa‐miR‐23a‐3p.^29^ The difference in expression values between these 2 miRNAs provides a good measure of the haemolysis degree, with values >5 suggesting erythrocyte miRNA contamination.^29^ The assay cut‐off was 35 cycles, and miRNAs expressed in at least 20% of the total sample.^30^

The miRNAs with complete data were used for the global mean method for normalization of the data, since this was found to be the most stable normalizer.^31^

#### 2.4.4 miRNA target prediction and functional enrichment analysis

Potential targets for the selected miRNAs were searched using miRWalk 3.0 (http://zmf.umm.uni-heidelberg.de/apps/zmf/mirwalk2/, accessed 30th May 2019). Statistical significances were reported after the Benjamini‐Hochberg (q‐value) correction for multiple comparisons. To better understand the biological relevance of the miRNAs target genes, a network analysis was executed using PathDIP (accessed 30th May 2019).^32^ The Jaccard similarity coefficient (JC) was implemented to elucidate functional similarities between dysregulated miRNAs. This criterion was adopted to investigate the similarity of miRNAs in a pairwise manner, both in terms of their target genes and enriched pathways.

### 2.5 Statistical analysis

Normal distribution was tested using the Kolmogorov‐Smirnov and Shapiro‐Wilk tests. Variables with normal distribution are showed as mean ± SD. Variables with skewed distribution were log‐transformed prior to analysis and were presented as median (25th–75th percentiles). Categorical data are shown as percentages. Clinical and laboratory characteristics and miRNA expressions were compared among groups using Student's *t* test or chi‐square tests, as appropriate. Correlations analyses were performed using Pearson's correlation tests. To assess the potential of individual miRNAs or groups of miRNAs in discriminating responders and non‐responders individuals, receiver operating characteristics (ROC) curves were generated, and areas under the curves (AUC) were calculated. Statistical analyses were done using the SPSS statistical package (v20.0) for Windows (SPSS Inc).

## 3 RESULTS

### 3.1 Clinical and laboratory characteristics of the individuals included in the study

Baseline clinical and laboratory characteristics of the participants are shown in Table 1. First, subjects with obesity and eutrophic individuals were compared for basal features. Second, the subjects with obesity were categorized into two intervention groups as follows: those following a moderately high‐protein diet (diet 1) and those following a low‐fat diet (diet 2).

**Table 1:** Baseline characteristics of study participants

Characteristic	All subjects with obesity (n = 78)	Moderately high‐protein diet (diet 1, n = 38)		Low‐fat diet (diet 2, n = 40)		Normal weight subjects (n = 25)	Diet 1 vs Diet 2	Responders vs NR
		Responders (n = 20)	Non‐responders (n = 18)	Responders (n = 20)	Non‐responders (n = 20)		*P* *	*P* *
Age (years)	46.6 ± 9.4	49.5 ± 8.6^b^	42.3 ± 9.3^b^	49.4 ± 6.9	44.9 ± 11.6	44.4 ± 9.1	.780	.030
Gender (% male)	36.1	42.9	57.1	54.2	45.8	40.0	.831	.348
BMI (kg/m^2^)	32.9 ± 2.4^a^	33.1 ± 2.2	32.9 ± 2.2	33.1 ± 2.5	32.4 ± 1.7	18.6 ± 2.1^a^	.941	.352
WC (cm)	104.9 ± 10.2^a^	107.1 ± 10.8	105.6 ± 11.3	105.1 ± 10.3	101.8 ± 8.2	75.1 ± 7.6^a^	.231	.328
FPG (mg/dL)	97.5 ± 11.9^a^	95.7 ± 11.6	98.1 ± 13.1	102.8 ± 12.5^c^	93.7 ± 10.3^c^	85.3 ± 6.9^a^	.666	.266
TC (mg/dL)	222.5 ± 40.1^a^	227.5 ± 42.9	219.4 ± 37.7	224.9 ± 38.3	216.3 ± 43.5	192.6 ± 37.1^a^	.691	.311
HDL‐c (mg/dL)	54.3 ± 14.0	56.1 ± 12.8	50.3 ± 12.1	53.5 ± 15.6	56.1 ± 14.6	61.6 ± 12.7	.501	.466
TG (mg/dL)	87.5 (63.5‐124.0)^a^	79.0 (69.0‐130.0)	88.5 (63.3‐140.5)	100.0 (76.0‐156.0)	77.0 (61.0‐110.0)	63.0 (41.0‐92.0)^a^	.513	.727
HOMA‐IR index	1.7 (1.1‐2.8)^a^	1.3 (1.0‐2.5)	2.0 (1.5‐2.8)	1.5 (1.1‐2.6)	1.7 (1.1‐2.8)	0.7 (0.4‐1.0)^a^	.970	.373
TyG index	8.4 ± 0.5^a^	8.3 ± 0.5	8.4 ± 0.6	8.5 ± 0.5^c^	8.2 ± 0.4^c^	7.8 ± 0.4^a^	.804	.417
Adiponectin (ng/mL)	10.9 (7.9‐13.5)	11.4 (8.2‐14.1)	10.4 (8.7‐13.5)	7.9 (6.4‐12.8)	11.3 (9.6‐13.3)	12.2 (9.3‐15.7)	.847	.662
Insulin (mU/L)	6.8 (4.7‐11.5)^a^	5.3 (4.5‐8.9)	9.1 (6.5‐13.7)	6.2 (4.3‐10.9)	8.1 (4.4‐10.4)	3.22 (2.9‐4.8)^a^	.943	.179
Leptin (ng/mL)	33.1 (17.2‐46.9)^a^	26.7 (18.4‐45.2)	37.7 (21.3‐47.1)	25.5 (8.7‐40.0)	36.8 (17.1‐49.8)	4.9 (2.1‐11.8)^a^	.619	.319
Body composition								
Fat mass (%)	34.7 ± 6.5^a^	35.3 ± 3.6	36.0 ± 3.6	33.6 ± 7.7	34.1 ± 7.4	13.7 ± 5.8^a^	.309	.622
Lean mass (%)	57.1 ± 11.8^a^	55.4 ± 11.2	59.1 ± 12.9	58.8 ± 11.9	54.8 ± 10.9	47.7 ± 12.2^a^	.771	.964
METs (kcal/kg/h)	17.0 (7.5‐27.0)^a^	19.6 (7.8‐26.0)	12.5 (6.4 ‐ 22.9)	20.9 (13.5‐34.9)	15.3 (7.2‐28.9)	33.2 (20.0‐44.4)^a^	.351	.294

Note

Variables are shown as mean ± SD, median (25th–75th percentiles) or %, as appropriate.

Abbreviations: BMI, body mass index; FPG, fasting plasma glucose; HDL‐c, high‐density lipoprotein cholesterol; HOMA‐IR, homeostatic model assessment‐insulin resistance; METs, metabolic equivalents; NR, non‐responders; TC, total cholesterol; TG, triglycerides; TyG, triglycerides glucose index; WC, waist circumference.

a Significant difference between subjects with obesity and individuals without obesity.

b Significant difference between responders and non‐responders to diet 1.

c Significant difference between responders and non‐responders to diet 2.

* P values were computed using chi‐square or Student *t* test, as appropriate.

As expected, subjects with obesity had higher waist circumference, fasting plasma glucose, total cholesterol and triglyceride levels compared to normal weight individuals (Table 1). Additionally, individuals with obesity also presented increased quantities of insulin, leptin, and HOMA‐IR and lower levels of METs compared to normal weight subjects (Table 1).

There were no differences in any baseline characteristic between the two dietary intervention sets. Moreover, comparing responders and non‐responders independently of diet, the only difference is regarding age, non‐responders by younger than responders (44.1 ± 10.4 vs 49.4 ± 7.7, *P* = .030). Likewise, non‐responders to diet 1 were younger than responders (*P* = .040). Responders to diet 2 showed increased levels of glucose and TyG index compared to non‐responders (*P* = .029 and *P* = .046, respectively), as shown in Table 1.

Changes in anthropometric and metabolic parameters due to the two dietary interventions are described in Table 2. As expected, after the 16‐week intervention, both diets induced improvements in anthropometric parameters and metabolic profile. However, no differences in weight loss, glucose metabolism or lipid profile were found between the two diets (Table 2). In the same way, responders to both dietary strategies also improved anthropometric and laboratorial characteristics. Additionally, no interactions were found among the analysed characteristics, dietary groups and response profile (Table 2).

**Table 2:** Changes in anthropometric characteristics and metabolic profile after 16‐week dietary intervention

Characteristic	Moderately high‐protein diet (diet 1; n = 38)				Low‐fat diet (diet 2; n = 40)				Diet 1 vs Diet 2	Responders vs NR	Interaction (Diet vs Response)
	All	Responders	Non‐responders	*P* *	All	Responders	Non‐responders	*P* *	*P* *	*P* *	*P* **
ΔWeight (kg)	−9.3	−14.1	−4.4	.0001	−9.4	−14.2	−3.7	.0001	.913	.0001	.452
Δ BMI (kg/m^2^)	−3.3	−5.1	−1.5	.0001	−3.4	−5.1	−1.3	.0001	.892	.0001	.662
Δ WC (cm)	−8.9	−13.9	−4.1	.0001	−9.4	−13.4	−4.4	.0001	.723	.0001	.633
Δ Fat mass (%)	−7.3	−11.1	−3.4	.0001	−7.2	−10.9	−3.6	.0001	.943	.0001	.625
Δ FPG (mg/dL)	−4.1	−6.1	−2.1	.173	−5.8	−9.7	−2.6	.018	.405	.007	.435
Δ TC (mg/dL)	−18.5	−34.7	−1.4	.001	−25.9	−39.2	−17.4	.023	.299	.0001	.375
Δ TG (mg/dL)	−17.5	−34.9	0.9	.013	−12.7	−31.9	3.9	.007	.630	.0001	.998
Δ Insulin (mlU/L)	−2.9	−3.5	−2.3	.440	−3.4	−4.0	−3.5	.674	.611	.356	.776
Δ Leptin (ng/mL)	−14.8	−25.1	−3.8	.008	−18.0	−24.9	−13.4	.117	.539	.002	.355
Δ HOMA‐IR index	−0.8	−0.9	−0.7	.602	−0.9	−1.2	−0.9	.410	.650	.334	.894
Δ TyG index	−0.2	−0.4	−0.04	.001	−0.2	−0.4	0.02	.002	.659	.0001	.850

Note

Variables are shown as difference of baseline and after 16‐week of intervention. Data are shown as mean ± SD, median (25th–75th percentiles) or %, as appropriate.

Abbreviations: BMI, body mass index; FPG, fasting plasma glucose; HDL‐c, high‐density lipoprotein cholesterol; HOMA‐IR, homeostatic model assessment‐insulin resistance; NR, non‐responders; TC, total cholesterol; TG, triglycerides; TyG, triglycerides glucose index; WC, waist circumference.

* *P* values were computed using Student's *t* test.

** Interactions among the characteristic, the diet group and the response to diet group were performed using ANOVA two‐way test (Univariate Analysis of Variance).

Interestingly, responders to low‐fat diet reduced leptin levels more than non‐responders to this diet. However, the same was not evidenced between responders and non‐responders to diet 1. Considering response pattern to diet 2, non‐responders showed a lower reduction in glucose levels than the responders, whereas this outcome was not found for the diet 1 (Table 2).

Regarding dietary consumption, the individuals randomized to diet 1 or diet 2 did not significantly differ in the dietary pattern at baseline, reporting no differences in the percentage of carbohydrates, fat, protein or fibre before the dietary intervention (*P* > .05). In the same way, subjects classified as responders and non‐responders, independently of the diet, also had comparable dietary patterns at baseline.

### 3.2 Quality control of miRNA expression

RNA spike‐in expressions presented low variation in Cq among samples in RNA isolation and cDNA synthesis, demonstrating that extraction, reverse transcription, and qPCR were effective and none of the samples contained inhibitors. Moreover, the UniSp5 was expressed in all analysed samples, demonstrating that miRNAs expressed at low levels were not lost during isolation. The ratio between miR‐451a and miR‐23a‐3p ranked between 5 and −1, showing that the samples were not affected by haemolysis. Generally, these results show a good and similar level of sample quality and reproducibility of the miRNA profiling processes.

### 3.3 Plasma microRNA expression

The expression of 86 target miRNAs was evaluated in plasma of responders and non‐responders of the two diets, and in normal weight individuals. Of these 86 miRNAs, 61 were expressed in at least 20% of the samples with Cq values ≥ 35 (Figure 1). First of all, 26 miRNAs were differentially expressed between subjects with obesity and normal weight individuals (Table S2 and Figure S1). Moreover, out of the 26 miRNAs, 9 were also associated with the response to a low‐fat diet (diet 2), but not with the response to a moderately high‐protein diet (Table 3).

**Table 3:** MicroRNAs differently expressed in responders and non‐responders to the two different diets

microRNAs	Cases with obesity vs controls without obesity		Moderately high‐protein diet (diet 1) Responders vs Non‐responders		Low‐fat diet (diet 2) Responders vs Non‐responders		All cases responders vs non‐responders		Interaction (miRNA, diet and response)
	Fold change	*P* *	Fold change	*P* *	Fold change	*P* *	Fold change	*P* *	*P* **
miR‐130a‐3p	0.40	.005	1.36	.871	0.67	.032	0.75	.430	.100
miR‐140‐3p	0.27	.0001	1.50	.814	0.80	.052	0.93	.197	.883
miR‐142‐5p	0.42	.002	0.88	.153	0.90	.035	0.81	.790	.022
miR‐144‐5p	0.19	.0001	2.33	.393	0.60	.025	1.10	.380	.772
miR‐148a‐3p	0.40	.006	1.50	.454	0.75	.051	1.20	.598	.955
miR‐15a‐5p	0.33	.022	2.00	.265	0.67	.021	1.67	.687	.677
miR‐22‐3p	0.39	.012	2.00	.313	2.10	.009	1.51	.445	.021
miR‐221‐3p	0.38	.004	1.50	.631	0.75	.046	1.08	.095	.235
miR‐29c‐3p	0.30	.007	1.10	.851	0.50	.035	1.57	.313	.732

Note

Data are shown as median (25th–75th percentiles) of n‐fold values. FC: fold change, FC values lower than 1 represents down‐regulation, and FC values higher than 1 represents up‐regulation in miRNA expression in responders compared to non‐responders.

* *P* values were obtained using Student *t* test using the log‐transformed variable.

** Interactions among the microRNA expression, the diet composition and the response to diet group were performed using ANOVA two‐way test (Univariate Analysis of Variance).

Comparing responders with non‐responders independently of the dietary intervention, no differences in miRNA expression were found between the two groups (Table S3). In the same way, there were no differences in miRNA expression between responders and non‐responders to the moderately high‐protein diet (Table 3 and Table S3). Instead, nine miRNAs were differentially expressed in responders compared to non‐responders to diet 2; however, 2 miRNAs (miR‐140‐3p and miR‐148a‐3p) did not reach formal statistical significance (Table 3 and Table S3). Then, 7 miRNAs (miR‐130a‐3p, miR‐142‐5p, miR‐144‐5p, miR‐15a‐5p, miR‐22‐3p, miR‐221‐3p and miR‐29c‐3p) were significantly differentially expressed in responders compared to non‐responders to diet 2 (Table 3). Interestingly, an interaction among 2 miRNAs, dietary group and response to diet was found [miR‐142‐5p (*r* ^2^ = .210, *P* = .022) and miR‐22‐3p (*r* ^2^ = .174, *P* = .021); Table 3].

**Figure 2:**
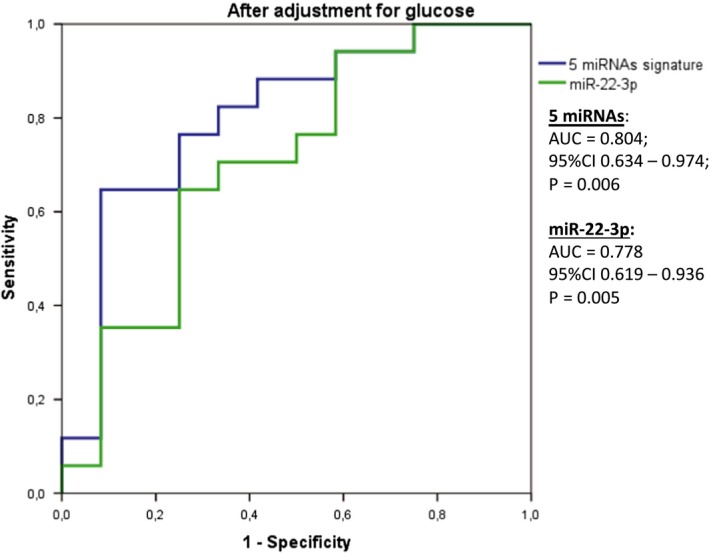
ROC curve to discriminate responders and non‐responders to diet 2 using 7 miRNAs (miR‐130a‐3p, miR‐142‐5p, miR‐144‐5p, miR‐15a‐5p, miR‐22‐3p, miR‐221‐3p and miR‐29c‐3p) and only miR‐22‐3p

The accuracy of single miRNAs and the combination of miRNA signatures to discriminate between responders and non‐responders to diet 2 were assessed by ROC analyses (Table 4). Considered individually, miR‐130b‐3p, miR‐142‐5p, miR‐144‐5p, miR‐221‐3p and miR‐22‐3p showed statistically significant AUC scores (0.729, 0.712, 0.714, 0.729 and 0.724); however, the AUC scores for miR‐15a‐5p and miR‐29c‐3p did not reach formal statistical significance (AUC = 0.678, *P* = .072; and AUC = 0.681, *P* = .073, respectively) (Table 4). The seven miRNA signature showed the strongest predictive value to discriminate responders from non‐responders (AUC = 0.770; 95% CI 0.594‐0.945; *P* = .015), demonstrating that the miRNA signature has a good performance to predict the response to diet. Additionally, the association was stronger after adjustment for basal glucose levels (AUC = 0.809; 95% CI 0.652‐0.966; *P* = .005) (Figure 2). Moreover, the adherence to the diet did not differ between responders and non‐responders to the low‐fat diet (*P* > .05).

**Table 4:** ROC curve analysis and logistic regression of microRNA differentially expressed in responders and non‐responders to a low‐fat diet

microRNA	Unadjusted		Adjusted^b^	
	AUC (95% CI); *P*	OR per 1‐SD (95% CI); *P* a	AUC (95% CI); *P*	OR per 1‐SD (95% CI); *P* a
miR‐130a‐3p	0.726 (0.555‐0.896); .025	4.378 (1.010‐18.969); .048	0.774 (0.610‐0.939); .006	3.907 (0.881‐17.321); .073
miR‐142‐5p	0.712 (0.533‐0.891); .035	3.729 (1.017‐13.674); .047	0.757 (0.585‐0.928); .011	3.191 (0.819‐12.435); .095
miR‐144‐5p	0.714 (0.535‐0.892); .034	3.018 (1.090‐8.356); .033	0.750 (0.581‐0.919); .013	2.697 (0.956‐7.606); .061
miR‐15a‐5p	0.678 (0.496‐0.860); .072	4.010 (0.970‐16.579); .055	0.752 (0.586‐0.917); .011	3.546 (0.818‐15.377); .091
miR‐22‐3p	0.724 (0.555‐0.893); .024	0.161 (0.033‐0.795); .025	0.778 (0.619‐0.936); .005	0.181 (0.084‐0.947); .043
miR‐221‐3p	0.729 (0.558‐0.900); .023	4.075 (0.909‐18.264); .066	0.760 (0.590‐0.930); .010	3.298 (0.750‐14.501); .114
miR‐29c‐3p	0.681 (0.495‐0.866); .073	3.988 (0.912‐17.445); .066	0.760 (0.585‐0.936); .010	3.784 (0.784‐18.271); .098

Note

The non‐responder group is the reference group.

a Effect measures are expressed as the ORs per SD increase of normalized relative miRNA level.

b Multivariable analyses were adjusted for basal glucose levels.

**Figure 3:**
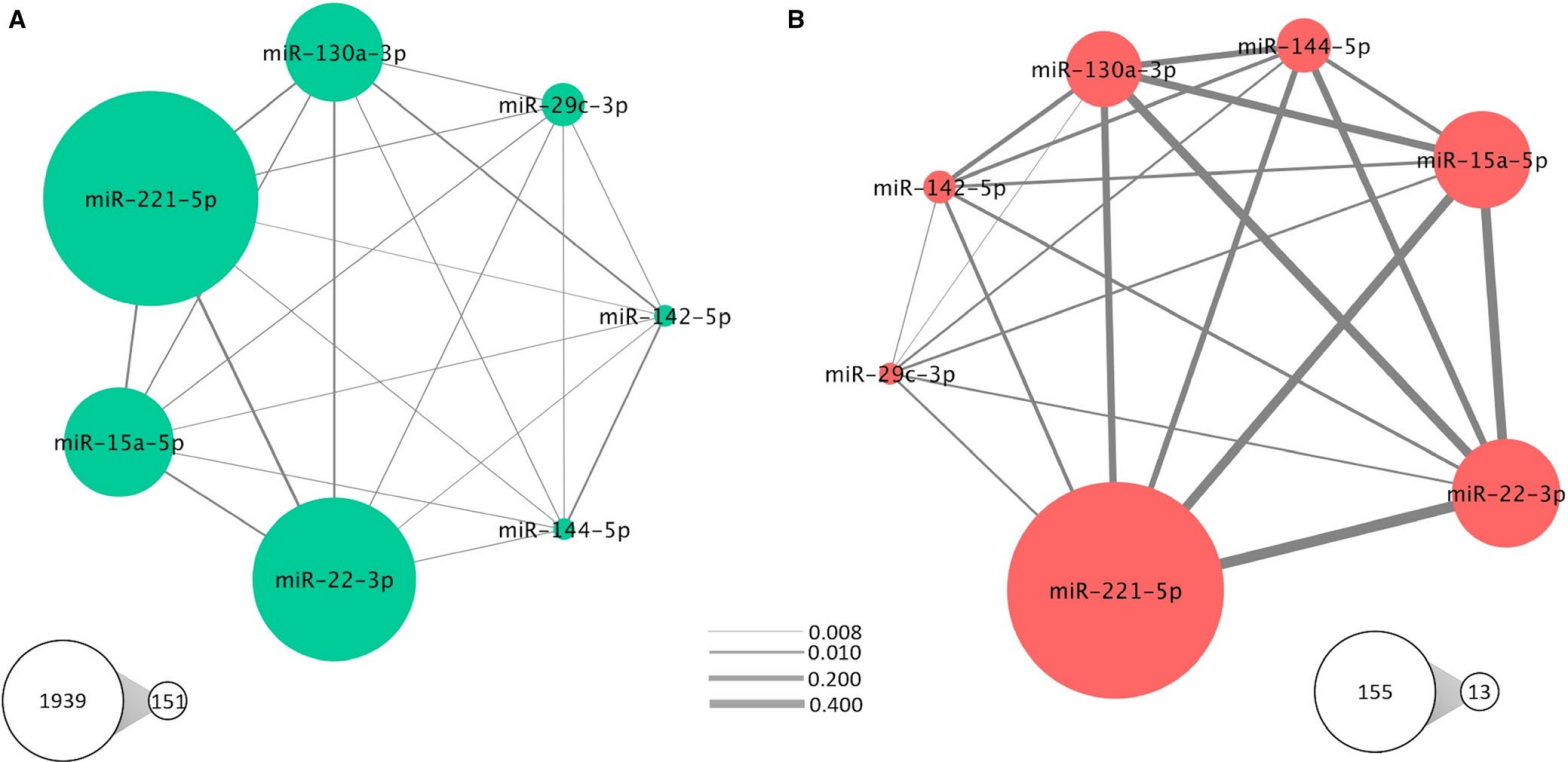
Jaccard similarity coefficients (JC) calculated in terms of number of target genes (A) and in number of pathways (B). The size of nodes represents number of targets or pathways for each miRNA and the edge width is proportional to the overlap between miRNA targets or pathways computed by JC

In univariate analyses, the ORs for the risk of non‐responders to diet 2 for a 1‐SD increase in the expression of miR‐130a‐3p was 4.378 [95% CI 1.010‐18.969; *P* = .048), for miR‐142‐5p was 3.729 [(95% CI 1.017‐13.674; *P* = .047)], and for miR‐144‐5p was 3.018 [(95% CI 1.017‐13.674; *P* = .047)]. Contrariwise, a 1‐SD increase in the miR‐22‐3p expression was associated with a decrease in the risk of non‐responding to diet 2 [OR = 0.161 (95% CI 0.033‐0.795; *P* = .025)] (Table 4). However, after adjustment for basal glucose levels, the results only remained statistically significant for miR‐22‐3p [OR = 0.181 (0.084‐0.947); *P* = .043] (Table 4 and Figure 3).

### 3.4 Target gene prediction and pathway enrichment analysis of differentially expressed miRNAs

Target gene prediction of the 7 differently expressed miRNAs between responders and non‐responders to diet 2 (miR‐130a‐3p, miR‐142‐5p, miR‐144‐5p, miR‐15a‐5p, miR‐221‐3p, miR‐22‐3p and miR‐29c‐3p) was accomplished using distinct databases of miRNA‐gene interactions in miRWalk environment. Following this methodology, 4,680 unique genes were found as targets of these 7 miRNAs analysed together; however, only 1,355 genes were found to be regulated by 2 or more miRNAs (Table S4). Of these genes, 572 were retrieved from experimentally validated interactions, while 4,108 were computationally predicted miRNA‐mRNA interactions (Table S4).

Subsequently, the list of the target genes among the 7 miRNAs was compared by calculating the JC values in a pairwise mode (Figure 3A). The highest JC values were obtained for miR‐221‐5p compared to miR‐22‐3p (JC = 0.10) and miR‐15a‐5p (JC = 0.08). However, as suggested by the JC values, the overlaps in the number of target genes are very low, demonstrating that few genes are shared among the miRNAs of interest.

**Figure 4:**
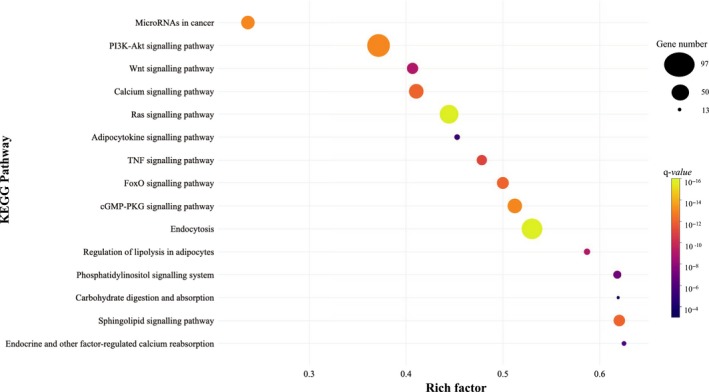
Significant KEGG pathways related to metabolism and potentially regulated by the seven miRNAs differentially expressed between responders and non‐responders to a low‐fat diet. The rich factor is the ratio of miRNAs target genes to the total gene number annotated in a certain pathway. The greater the rich factor, the greater the degree of pathway enrichment. The size and the colour of the dots represent the gene number and the range of the pathway's *q*‐value, respectively. *Q*‐values: *P* values corrected for multiple tests using the Benjamini‐Hochberg procedure. miRNA: microRNA

To further investigate the functional consequences of dysregulation of the 7 selected miRNAs, functional enrichment analyses were performed using KEGG Pathways in the PathDIP environment. A total of 81 pathways were significantly over‐represented (q‐values < 0.05) in the analysed lists of target genes. As expected, the results from pathway enrichment analysis based on predicted/validated targets of the 7 miRNAs showed a number of already known metabolism‐associated pathways, including regulation of lipolysis in adipocytes, sphingolipid, FoxO, TNF, Wnt and adipocytokine signalling pathways (Figure 4 and Table S5). Additionally, the similarity among the 7 miRNAs regarding the enriched pathways was also analysed using the pairwise JC and a substantial overlap in the list of modulated pathways were found for some pairs of miRNAs, especially among miR‐22‐3p, miR‐221‐3p and miR‐15a‐5p (Figure 3B). These findings may indicate that these miRNAs participate in shared pathways through distinct mechanisms and targets, having complementary roles in their modulation and function.

## 4 DISCUSSION

Recent studies have revealed the critical role of several miRNAs in the energy homeostasis by affecting the expression of genes and proteins involved in metabolic processes.^11^, ^25^ Some miRNAs have been implicated in the control of body weight, glucose homeostasis, insulin sensitivity and lipid metabolism,^31^, ^32^ suggesting that miRNAs could be used as therapeutic targets for the treatment of metabolic disorders, but also as predictive biomarkers.^11^

Circulating miRNAs are protected from RNAses in the extracellular environment especially through association with microvesicles, exosomes or proteins, thus participating in cell‐to‐cell communication.^33^, ^34^ Moreover, circulating miRNAs levels usually reflect a systemic response to an external stimulus and their expression measurements may uncover non‐invasive biomarkers for human diseases.^35^ Furthermore, some researchers have demonstrated that circulating miRNAs can be regulated by nutrients from diet or bioactive compounds, which could explain why some miRNAs changed their expression pattern in response to a specific diet.^36^ However, few studies have analysed the effects of nutrients on the regulation of circulating miRNAs under different metabolic conditions.

The results from the current study shown a marked dysregulation in the expression levels of 7 miRNAs (miR‐130a‐3p, miR‐142‐5p, miR‐144‐5p, miR‐15a‐5p, miR‐22‐3p, miR‐221‐3p and miR‐29c‐3p) in responders compared to non‐responders to a low‐fat diet. Generally, these 7 miRNAs are associated with various metabolic processes, including glucose and lipid metabolism,^37^, ^38^, ^39^ adipocyte development and adipose tissue physiology,^10^, ^25^ inflammation pathways 40 and weight gain.^41^ These findings suggest that circulating miRNAs could be used as biomarkers of the response to specific weight loss dietary interventions.

In addition to indicating a group of miRNAs predicting the response to a low‐fat diet, the present research also provides novel understandings into the complex molecular mechanisms involved in obesity and weight loss, revealing pathways that may be regulated by miR‐130a‐3p, miR‐142‐5p, miR‐144‐5p, miR‐15a‐5p, miR‐22‐3p, miR‐221‐3p and miR‐29c‐3p. These miRNAs potentially modulate the expression of genes participating in several pathways related to adiposity, metabolism and inflammation, such as regulation of lipolysis in adipocytes, sphingolipid, FoxO, TNF, Wnt and adipocytokine signalling.

Regarding miR‐22‐3p, 1‐SD increase in the relative expression of this miRNA was associated with a reduction on the possibility of a non‐response to low‐fat diet. Moreover, this miRNA was also associated with the percentage of weight loss in our sample. In this context, miR‐22 is known to directly target peroxisome proliferative activated receptor, gamma, coactivator 1 alpha (*Pgc‐1α*), peroxisome proliferator‐activated receptor α (*Pparα*) and Sirtuin 1 (*Sirt1*),^42^, ^43^ which are important genes involved in fatty acid metabolism, mitochondrial biogenesis and obesity. Most recently, it has been reported that miR‐22 is required for normal body weight gain in male mice,^44^ raising the possibility that miR‐22 may play a role in obesity.

In addition to miR‐22‐3p, an interaction between diet composition, diet response and miR‐142‐5p expression was also reported. Supporting our results, high levels of miR‐142 have been associated with an increased risk of significant weight gain during a 5‐year follow‐up of a group of healthy Mexican‐American women.^41^ Furthermore, miR‐142 was up‐regulated in adipose tissue of mice after long‐term (5‐month) high‐fat diet‐induced obesity compared to standard‐fed counterparts.^45^

Moreover, compelling evidence suggests that circulating miRNAs are associated with obesity.^14^, ^41^, ^46^, ^47^ In this context, a set of 26 miRNAs that were mostly described as associated with obesity in a recently systematic review study 48 were found differently expressed in subjects with obesity compared to non‐obese individuals. In agreement with our results, miR‐21‐5p, miR‐103a and miR‐221‐3p were also found as down‐regulated in blood sample of subjects with obesity in a meta‐analysis study.^49^ Additionally, the miRNAs that were dysregulated in obesity in the present study are associated with various metabolic processes, such as impaired glucose tolerance,^47^ maintenance of the pancreatic beta cell mass,^50^ adipocyte development and adipose tissue physiology,^10^, ^25^ inflammation pathways 40 and cardiomyocyte survival y.^51^

Some strengths and limitations of our study should be considered. As strengths, a very‐well characterized sample from a randomized clinical trial was analysed. Moreover, we used several quality controls for miRNA extraction, cDNA synthesis and PCR process. Even though these methods are powerful, this evaluation has some limitations. First, the reduced sample size which could lead to lack of power to detect small differences in miRNA expression between groups. Second, the miRNA expression analyses were done in uniplicate. Third, our approach identifies correlations and not causal relationships. Nevertheless, this strategy allowed generating potential interactions between miRNAs expression and diet response. Fourth, even though a hypothesis driving approach was performed, the possibility of type I or type II errors due to multiple comparisons cannot be excluded. Therefore, these limitations should be considered when interpreting the results.

In conclusion, this research provides novel information showing that the circulating levels of 7 miRNAs differ between responders and non‐responders to a low‐fat diet intervention. Additionally, these miRNAs regulate genes implicated in important pathways related to metabolism, adiposity, inflammation and obesity development. Moreover, our findings demonstrate the potential use of circulating miRNAs as predictive biomarkers of weight loss to a specific dietary intervention, which may help to the implementation of precision nutrition. However, further research with larger cohorts of participants and longer duration of intervention will be important for determining whether these miRNAs participate in the regulation of the cellular mechanisms that control weight loss and therefore become accurate biomarkers for obesity management.

## CONFLICT OF INTEREST

The authors declared no conflict of interest.

## AUTHORS' CONTRIBUTIONS

TSA designed the study, analysed and interpreted the data, and drafted the manuscript. JIR designed the study, interpreted the data and critically reviewed the manuscript. FIM designed and supervised the study, interpreted the data and critically reviewed the manuscript. J.AM. designed and supervised the study, interpreted the data and critically reviewed the manuscript. All authors approved the final version and agreed to be accountable for all aspects of the work regarding accuracy and integrity aspects.

## Data Availability

All data generated or analysed during this study are included in this published article in and its supplementary files. Requests for material should be made to the corresponding author.
