# Supplementary figures and images for: Circulating adiposity‐related microRNAs as predictors of the response to a low‐fat diet in subjects with obesity
Source: J Cell Mol Med. 2020 Jan 22;24(5):2956–67. doi: 10.1111/jcmm.14920 (PMC7077528; doi:10.1111/jcmm.14920)

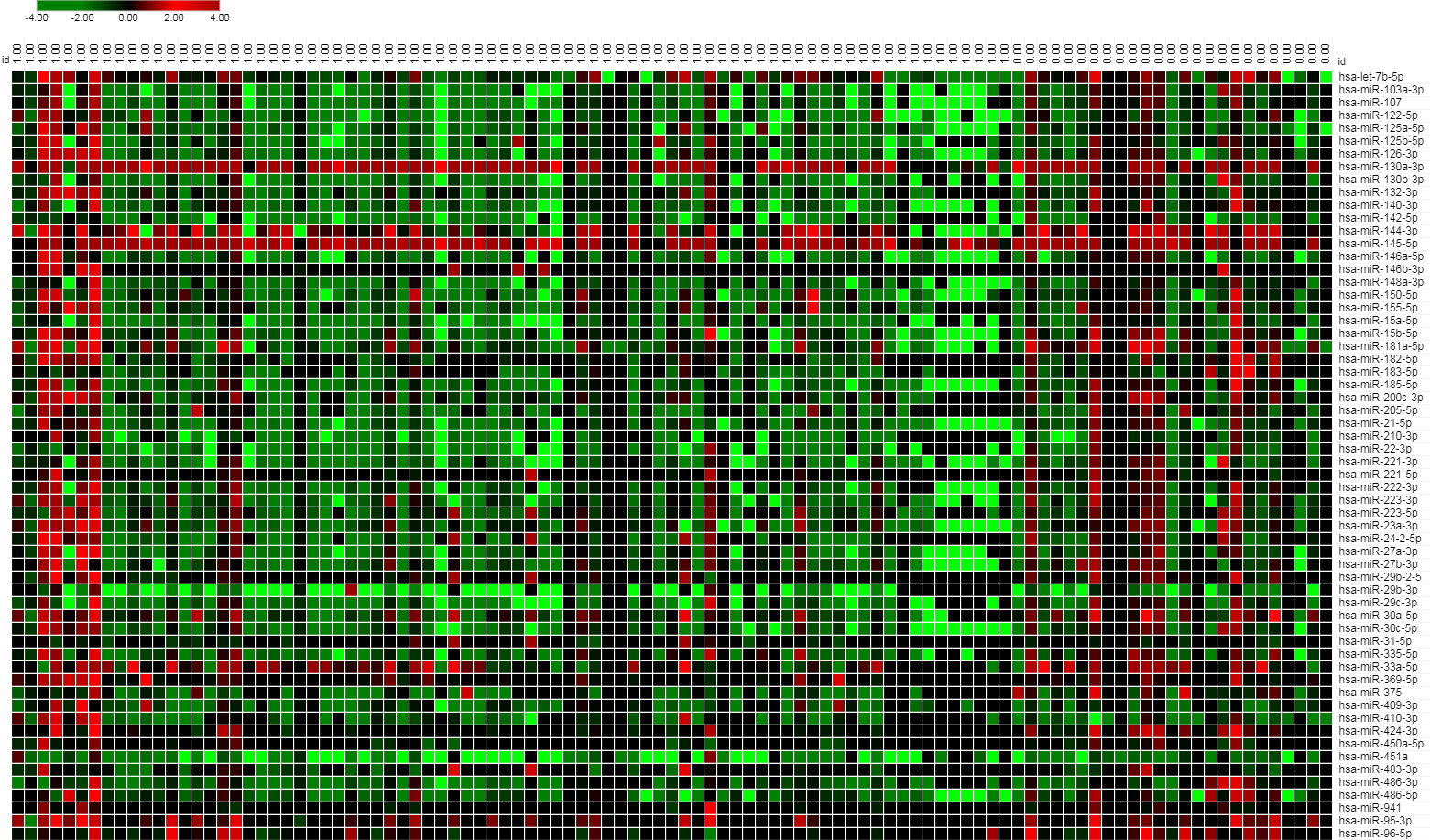

Supplement: Supplementary file 1 [file JCMM-24-2956-s001.tiff]
