# Supplementary material for: Circulating adiposity‐related microRNAs as predictors of the response to a low‐fat diet in subjects with obesity
Source: J Cell Mol Med. 2020 Jan 22;24(5):2956–67. doi: 10.1111/jcmm.14920 (PMC7077528; doi:10.1111/jcmm.14920)
Supplement: Supplementary file 2 [file JCMM-24-2956-s002.docx]

**Supplementary table 1.** Assay reference numbers for the 96 analyzed miRNAs included in the Pick-&-Mix microRNA PCR Panel, 384 well Ready-to-use, v5.

| microRNA ID | Corresponding LNA microRNA primer set (Prod No) |
| --- | --- |
| hsa-let-7b-5p | 204750 |
| hsa-miR-103a-3p | 204063 |
| hsa-miR-107 | 204468 |
| hsa-miR-1203 | 204414 |
| hsa-miR-122-5p | 205664 |
| hsa-miR-125a-5p | 204339 |
| hsa-miR-125b-5p | 205713 |
| hsa-miR-126-3p | 204227 |
| hsa-miR-130a-3p | 204658 |
| hsa-miR-130b-3p | 204317 |
| hsa-miR-132-3p | 206035 |
| hsa-miR-138-5p | 206078 |
| hsa-miR-140-3p | 204304 |
| hsa-miR-142-5p | 204722 |
| hsa-miR-144-3p | 204754 |
| hsa-miR-145-5p | 204483 |
| hsa-miR-146a-3p | 204401 |
| hsa-miR-146a-5p | 204688 |
| hsa-miR-146b-3p | 204374 |
| hsa-miR-146b-5p | 204553 |
| hsa-miR-148a-3p | 205867 |
| hsa-miR-150-5p | 204660 |
| hsa-miR-155-3p | 204000 |
| hsa-miR-155-5p | 204308 |
| hsa-miR-15a-5p | 204066 |
| hsa-miR-15b-5p | 204243 |
| hsa-miR-181a-5p | 206081 |
| hsa-miR-182-5p | 206070 |
| hsa-miR-183-5p | 206030 |
| hsa-miR-185-5p | 206037 |
| hsa-miR-196a-5p | 204386 |
| hsa-miR-200a-5p | 206063 |
| hsa-miR-200b-5p | 204144 |
| hsa-miR-200c-3p | 204482 |
| hsa-miR-200c-5p | 204479 |
| hsa-miR-205-5p | 204487 |
| hsa-miR-208b-3p | 204636 |
| hsa-miR-210-3p | 204333 |
| hsa-miR-21-5p | 204230 |
| hsa-miR-216a-5p | 204167 |
| hsa-miR-221-3p | 204532 |
| hsa-miR-221-5p | 204032 |
| hsa-miR-222-3p | 204551 |
| hsa-miR-223-3p | 205986 |
| hsa-miR-223-5p | 204529 |
| hsa-miR-22-3p | 204606 |
| hsa-miR-23a-3p | 204772 |
| hsa-miR-24-2-5p | 204187 |
| hsa-miR-27a-3p | 206038 |
| hsa-miR-27b-3p | 205915 |
| hsa-miR-29b-2-5p | 204208 |
| hsa-miR-29b-3p | 204679 |
| hsa-miR-29c-3p | 204729 |
| hsa-miR-30a-5p | 205695 |
| hsa-miR-30c-5p | 204783 |
| hsa-miR-31-5p | 204236 |
| hsa-miR-335-5p | 204151 |
| hsa-miR-33a-5p | 205690 |
| hsa-miR-33b-5p | 205860 |
| hsa-miR-369-5p | 206014 |
| hsa-miR-375 | 204362 |
| hsa-miR-409-3p | 204358 |
| hsa-miR-410-3p | 204042 |
| hsa-miR-412-3p | 204460 |
| hsa-miR-424-3p | 205918 |
| hsa-miR-450a-5p | 206085 |
| hsa-miR-483-3p | 204012 |
| hsa-miR-486-3p | 204107 |
| hsa-miR-486-5p | 204001 |
| hsa-miR-488-3p | 204469 |
| hsa-miR-519d-3p | 204062 |
| hsa-miR-519e-5p | 204029 |
| hsa-miR-541-3p | 204276 |
| hsa-miR-548d-5p | 205907 |
| hsa-miR-557 | 204346 |
| hsa-miR-572 | 204696 |
| hsa-miR-612 | 204068 |
| hsa-miR-615-3p | 204453 |
| hsa-miR-659-3p | 204199 |
| hsa-miR-758-3p | 204353 |
| hsa-miR-892a | 204092 |
| hsa-miR-935 | 205579 |
| hsa-miR-941 | 204574 |
| hsa-miR-95-3p | 204288 |
| hsa-miR-96-5p | 204417 |
| UniSp2 | 203950 |
| UniSp4 | 203953 |
| UniSp3 IPC |  |
| UniSp5 | 203955 |
| UniSp6 |  |
| cel-miR-39-3p | 203952 |
| Blank |  |
| SNORD38B | 203901 |
| SNORD49A | 203904 |
| U6 snRNA | 203907 |
