# Supplementary material for: Circulating adiposity‐related microRNAs as predictors of the response to a low‐fat diet in subjects with obesity
Source: J Cell Mol Med. 2020 Jan 22;24(5):2956–67. doi: 10.1111/jcmm.14920 (PMC7077528; doi:10.1111/jcmm.14920)
Supplement: Supplementary file 3 [file JCMM-24-2956-s003.docx]

**Supplementary table 2.** Twenty-six microRNAs differently expressed between subjects with obesity compared to eutrophic individuals.

| **microRNA** | **Subjects with obesity (n= 78)** | **Eutrophic individuals (n= 25)** | **P-*value**** | **q-*value***** |
| --- | --- | --- | --- | --- |
| miR-103a-3p | 0.169 (0.057 – 0.443) | 0.567 (0.263 – 1.426) | 0.006 | 0.020 |
| miR-107 | 0.201 (0.067 – 0.520) | 0.614 (0.309 – 1.386) | 0.014 | 0.038 |
| miR-130a-3p | 19.321 (6.029 – 32.947) | 47.904 (31.421 – 79.317) | 0.005 | 0.004 |
| miR-130b-3p | 0.208 (0.096 – 0.433) | 0.442 (0.221 – 1.426) | 0.003 | 0.015 |
| miR-140-3p | 0.237 (0.076 – 0.578) | 0.872 (0.548 – 1.561) | 0.0001 | 0.0012 |
| miR-142-5p | 0.118 (0.036 – 0.214) | 0.279 (0.162 – 0.941) | 0.002 | 0.007 |
| miR-144-3p | 1.308 (0.276 – 2.872) | 6.903 (2.036 – 11.542) | 0.0001 | 0.0012 |
| miR-148a-3p | 0.259 (0.096 – 0.479) | 0.647 (0.212 – 1.135) | 0.006 | 0.019 |
| miR-181a-5p | 0.549 (0.229 – 1.107) | 1.633 (0.369 – 3.021) | 0.006 | 0.008 |
| miR-183-5p | 0.374 (0.244 – 0.649) | 0.775 (0.476 – 1.928) | 0.001 | 0.009 |
| miR-185-5p | 0.198 (0.083 – 0.480) | 0.665 (0.288 – 1.272) | 0.034 | 0.040 |
| miR-200c-3p | 0.540 (0.257 – 1.118) | 1.001 (0.454 – 2.161) | 0.037 | 0.044 |
| miR-205-5p | 0.163 (0.122 – 0.559) | 0.711 (0.319 – 1.322) | 0.005 | 0.020 |
| miR-21-5p | 0.257 (0.137 – 0.615) | 0.559 (0.261 – 1.238) | 0.036 | 0.041 |
| miR-210-3p | 0.110 (0.071 – 0.245) | 0.503 (0.117 – 0.672) | 0.030 | 0.078 |
| miR-221-3p | 0.212 (0.065 – 0.454) | 0.555 (0.268 – 1.549) | 0.004 | 0.013 |
| miR-222-3p | 0.311 (0.158 – 0.712) | 0.859 (0.470 – 1.396) | 0.005 | 0.019 |
| miR-15a-5p | 0.118 (0.060 – 0.322) | 0.356 (0.155 – 0.877) | 0.022 | 0.054 |
| miR-22-3p | 0.126 (0.056 – 0.286) | 0.320 (0.127 – 1.027) | 0.012 | 0.034 |
| miR-29c-3p | 0.137 (0.068 – 0.350) | 0.453 (0.202 – 1.269) | 0.007 | 0.020 |
| miR-30a-5p | 0.629 (0.294 – 1.347) | 1.426 (0.515 – 2.062) | 0.043 | 0.048 |
| miR-30c-5p | 0.274 (0.093 – 0.625) | 0.694 (0.235 – 1.462) | 0.042 | 0.050 |
| miR-33a-5p | 1.268 (0.573 – 2.425) | 4.54 (1.009 – 23.596) | 0.012 | 0.016 |
| miR-375 | 0.228 (0.124 – 0.609) | 0.765 (0.484 – 1.563) | 0.0001 | 0.002 |
| miR-424-3p | 0.892 (0.698 – 1.135) | 2.000 (0.834 – 3.127) | 0.016 | 0.030 |
| miR-486-3p | 0.301 (0.190 – 0.570) | 0.645 (0.238 – 1.326) | 0.004 | 0.009 |

Data are shown as median (25th–75th percentiles) of n-fold values.

*P-*values* were obtained using Student t test using the log-transformed variable. **P-*values* were corrected using Benjamini-Hochberg false discovery rate (FDR; q-*value*).
