# Supplementary material for: Circulating adiposity‐related microRNAs as predictors of the response to a low‐fat diet in subjects with obesity
Source: J Cell Mol Med. 2020 Jan 22;24(5):2956–67. doi: 10.1111/jcmm.14920 (PMC7077528; doi:10.1111/jcmm.14920)
Supplement: Supplementary file 4 [file JCMM-24-2956-s004.docx]

**Supplementary table 3.** The expression profile of 61 miRNAs with Cq values <35 in at least 20% of total sample broken down by the response to the two diets.

| **microRNA** | **Moderately-high-protein diet**  **(diet 1)**  **Responders *vs* Non-responders** | | **Low-fat diet**  **(diet 2)**  **Responders *vs* Non-responders** | | **All cases**  **Responders *vs* Non-responders** | |
| --- | --- | --- | --- | --- | --- | --- |
|  | **Fold-change** | **P*** | **Fold-change** | **P*** | **Fold-change** | **P*** |
| **hsa-let-7b-5p** | 0.82 | 0.128 | 0.76 | 0.640 | 0.98 | 0.621 |
| **hsa-miR-103a-3p** | 0.73 | 0.874 | 0.67 | 0.083 | 0.71 | 0.232 |
| **hsa-miR-107** | 1.15 | 0.961 | 0.75 | 0.092 | 0.82 | 0.313 |
| **hsa-miR-122-5p** | 1.52 | 0.493 | 0.35 | 0.143 | 0.68 | 0.865 |
| **hsa-miR-125a-5p** | 1.14 | 0.403 | 0.48 | 0.257 | 1.02 | 0.996 |
| **hsa-miR-125b-5p** | 1.05 | 0.639 | 1.94 | 0.914 | 1.08 | 0.753 |
| **hsa-miR-126-3p** | 1.33 | 0.254 | 0.43 | 0.117 | 1.27 | 0.646 |
| **hsa-miR-130a-3p** | 1.36 | 0.871 | 0.67 | **0.032** | 0.75 | 0.430 |
| **hsa-miR-130b-3p** | 1.15 | 0.779 | 0.86 | 0.135 | 1.09 | 0.604 |
| **hsa-miR-132-3p** | 0.80 | 0.969 | 0.61 | 0.637 | 0.89 | 0.673 |
| **hsa-miR-140-3p** | 1.50 | 0.814 | 0.80 | **0.052** | 0.93 | 0.197 |
| **hsa-miR-142-5p** | 0.88 | 0.153 | 0.90 | **0.035** | 0.81 | 0.790 |
| **hsa-miR-144-3p** | 2.33 | 0.393 | 0.60 | **0.025** | 1.10 | 0.772 |
| **hsa-miR-145-5p** | 0.89 | 0.957 | 0.95 | 0.652 | 1.03 | 0.882 |
| **hsa-miR-146a-5p** | 1.66 | 0.232 | 0.63 | 0.152 | 1.27 | 0.966 |
| **hsa-miR-146b-5p** | 0.65 | 0.908 | 0.67 | 0.367 | 0.98 | 0.611 |
| **hsa-miR-148a-3p** | 1.50 | 0.454 | 0.75 | **0.051** | 1.20 | 0.955 |
| **hsa-miR-150-5p** | 1.5 | 0.126 | 0.64 | 0.324 | 0.77 | 0.537 |
| **hsa-miR-155-5p** | 1.05 | 0.992 | 0.38 | 0.193 | 0.84 | 0.349 |
| **hsa-miR-15a-5p** | 2.0 | 0.265 | 0.67 | **0.021** | 1.67 | 0.677 |
| **hsa-miR-15b-5p** | 1.89 | 0.208 | 0.34 | **0.068** | 1.12 | 0.981 |
| **hsa-miR-181a-5p** | 0.82 | 0.689 | 0.54 | 0.445 | 0.87 | 0.827 |
| **hsa-miR-182-5p** | 1.34 | 0.691 | 0.64 | 0.812 | 0.75 | 0.933 |
| **hsa-miR-183-5p** | 1.07 | 0.699 | 1.18 | 0.269 | 1.09 | 0.387 |
| **hsa-miR-185-5p** | 1.13 | 0.691 | 0.49 | 0.180 | 1.31 | 0.730 |
| **hsa-miR-200c-3p** | 1.22 | 0.289 | 0.37 | 0.229 | 1.18 | 0.974 |
| **hsa-miR-205-5p** | 1.07 | 0.626 | 0.33 | 0.160 | 0.40 | 0.618 |
| **hsa-miR-210-3p** | 1.12 | 0.583 | 0.42 | 0.229 | 1.47 | 0.812 |
| **hsa-miR-21-5p** | 1.32 | 0.183 | 0.58 | 0.327 | 1.07 | 0.545 |
| **hsa-miR-22-3p** | 2.00 | 0.313 | 2.10 | **0.009** | 1.51 | 0.445 |
| **hsa-miR-221-3p** | 1.50 | 0.631 | 0.75 | **0.046** | 1.08 | 0.095 |
| **hsa-miR-221-5p** | 12.24 | 0.335 | 1.00 | 0.870 | 1.41 | 0.258 |
| **hsa-miR-222-3p** | 1.41 | 0.462 | 0.58 | 0.060 | 0.61 | 0.592 |
| **hsa-miR-223-3p** | 1.00 | 0.981 | 0.71 | 0.888 | 0.45 | 0.861 |
| **hsa-miR-223-5p** | 1.16 | 0.187 | 0.46 | 0.963 | 1.20 | 0.336 |
| **hsa-miR-23a-3p** | 1.13 | 0.354 | 0.78 | 0.992 | 1.12 | 0.482 |
| **hsa-miR-24-2-5p** | 2.00 | 0.217 | 0.55 | 0.686 | 0.90 | 0.274 |
| **hsa-miR-27a-3p** | 0.95 | 0.622 | 0.31 | 0.062 | 1.04 | 0.186 |
| **hsa-miR-27b-3p** | 0.95 | 0.946 | 0.77 | 0.636 | 1.15 | 0.915 |
| **hsa-miR-29b-3p** | 0.97 | 0.753 | 0.57 | 0.973 | 0.75 | 0.867 |
| **hsa-miR-29b-2-5p** | 1.39 | 0.522 | 1.24 | 0.232 | 1.32 | 0.157 |
| **hsa-miR-29c-3p** | 1.10 | 0.851 | 0.50 | **0.035** | 1.57 | 0.313 |
| **hsa-miR-30a-5p** | 0.86 | 0.712 | 0.64 | 0.436 | 0.80 | 0.417 |
| **hsa-miR-30c-5p** | 1.19 | 0.480 | 0.65 | 0.201 | 0.86 | 0.872 |
| **hsa-miR-31-5p** | 0.79 | 0.723 | 1.32 | 0.263 | 0.77 | 0.340 |
| **hsa-miR-335-5p** | 0.84 | 0.974 | 0.54 | 0.230 | 1.09 | 0.352 |
| **hsa-miR-33a-5p** | 0.86 | 0.749 | 0.55 | 0.093 | 0.79 | 0.212 |
| **hsa-miR-369-5p** | 2.39 | 0.310 | 1.42 | 0.399 | 1.62 | 0.144 |
| **hsa-miR-375** | 2.15 | 0.489 | 0.35 | 0.508 | 1.01 | 0.874 |
| **hsa-miR-409-3p** | 1.61 | 0.244 | 0.52 | 0.509 | 1.14 | 0.705 |
| **hsa-miR-410-3p** | 1.80 | 0.713 | 1.95 | 0.564 | 1.73 | 0.496 |
| **hsa-miR-424-3p** | 1.05 | 0.576 | 0.90 | 0.772 | 0.84 | 0.813 |
| **hsa-miR-450a-5p** | 0.46 | 0.354 | 0.69 | 0.871 | 0.73 | 0.368 |
| **hsa-miR-451a** | 1.4 | 0.803 | 1.00 | 0.961 | 1.33 | 0.779 |
| **hsa-miR-483-3p** | 1.30 | 0.406 | 0.58 | 0.370 | 0.78 | 0.216 |
| **hsa-miR-486-3p** | 1.27 | 0.963 | 0.50 | 0.062 | 0.70 | 0.272 |
| **hsa-miR-486-5p** | 1.13 | 0.520 | 0.69 | 0.982 | 0.88 | 0.634 |
| **hsa-miR-941-3p** | 1.20 | 0.999 | 1.77 | 0.913 | 1.12 | 0.938 |
| **hsa-miR-95-3p** | 0.75 | 0.562 | 0.55 | 0.789 | 0.68 | 0.441 |
| **hsa-miR-96-5p** | 0.19 | 0.188 | 1.14 | 0.286 | 0.89 | 0.986 |

Data are shown as median (25th – 75th percentiles) of n-fold values. *P *values* were computed using Student’s t-test with the log-transformed variable. FC: fold-change, FC values lower than 1 represents downregulation, and FC values higher than 1 represents upregulation in miRNA expression in responders compared to non-responders.
